# A New Use for the Appendix

**Published:** 1902-08-30

**Authors:** 


### A NEW USE FOR THE APPENDIX.

It is only now and again that a patient suffers from colitis in such an extreme degree as to make it desirable to perform right inguinal colostomy with the double object of diverting the stream of irritating fæces and of furnishing an opening by which medicated fluids may be introduced so as to flush the diseased surfaces. When, however, a case does require to be treated in this manner, then at last the otherwise useless appendix can be put to some useful purpose, for, according to Dr. Robert F. Weir,[1] of New York, instead of doing a right inguinal colostomy or even adopting the simpler and better plan of producing a cæcal fistula, the appendix may be brought up and fastened to the skin so as itself to form the track from the skin to the cavity of the cæcum. This is a noteworthy suggestion. Dr. Weir says that an artificial anus is more incontrollable when situated in the right than in the left iliac fossa from the difficulty of forming a "spur," and is always a very distressing measure to employ. Lately, however, it has been found that by simply making a fistulous opening in the cæcum and in this way thoroughly washing out the bowel of its fæces and then flushing it with a medicated solution one could accomplish a cure without employing the disgusting artificial anus. The plan adopted is that of making a cæcal fistula in the same way that Kader performs a gastrostomy—that is to say, by placing a rubber catheter of medium size in a small opening made in the bowel and then applying two or three rows of a purse-string suture, so as to invert the adjacent peritoneal coat. This causes a teat-like projection into the lumen of the bowel, which acts later as a valve, and prevents leakage to a marked degree. When it so acts the catheter need only be introduced once or twice a day for the necessary medications. In doing this operation upon a young man who had for three years been suffering from colitis (persistent diarrhœa with profuse loss of blood, by which he had been reduced to a condition of extreme anæmia), as the cæcum was exposed the appendix rose so suggestively into view that Dr. Weir determined to employ it to make the desired fistula. It was accordingly fastened to the skin and the rest of the wound closed. The tip of the appendix was then opened and a No. 12 English rubber catheter was passed, which entered freely into the cæcum. The freedom of the communication having thus been proved, the opening was closed beyond the level of the skin by a temporary ligature, which was, however, removed the next day when irrigation treatment was commenced. The outcome of this case, says Dr. Weir, has been a most happy one, not only as to a progress towards a cure by virtue of the irrigation, but also from the fact that the normal muscularity of the appendical wall has prevented any leakage from the, so to speak, normal fistula, which furthermore has not had the same tendency to close as had the walls of the peritoneally-formed Kader fistula. When the stage of normal defecation is reached, one need only plunge the point of a Paquelin cautery into the lumen of the fistula to cause its obliteration, or if its anchorage to the abdominal wall be considered inadvisable, the usual "interval" operation for the removal of the appendix can be performed.

[1] New York Medical Record.

